# Eigenproblem Versus the Load-Carrying Capacity of Hybrid Thin-Walled Columns with Open Cross-Sections in the Elastic Range

**DOI:** 10.3390/ma14133468

**Published:** 2021-06-22

**Authors:** Zbigniew Kolakowski, Andrzej Teter

**Affiliations:** 1 Department of Strength of Materials (K12), Faculty of Mechanical Engineering, Lodz University of Technology (TUL), Stefanowskiego 1/15, PL-90-924 Lodz, Poland; 2 Department of Applied Mechanics, Faculty of Mechanical Engineering, Lublin University of Technology (LUT), Nadbystrzycka 36, PL-20-618 Lublin, Poland; a.teter@pollub.pl

**Keywords:** multi-mode buckling, imperfection, compression, FEM, Koiter’s theory

## Abstract

The phenomena that occur during compression of hybrid thin-walled columns with open cross-sections in the elastic range are discussed. Nonlinear buckling problems were solved within Koiter’s approximation theory. A multimodal approach was assumed to investigate an effect of symmetrical and anti-symmetrical buckling modes on the ultimate load-carrying capacity. Detailed simulations were carried out for freely supported columns with a C-section and a top-hat type section of medium lengths. The columns under analysis were made of two layers of isotropic materials characterized by various mechanical properties. The results attained were verified with the finite element method (FEM). The boundary conditions applied in the FEM allowed us to confirm the eigensolutions obtained within Koiter’s theory with very high accuracy. Nonlinear solutions comply within these two approaches for low and medium overloads. To trace the correctness of the solutions, the Riks algorithm, which allows for investigating unsteady paths, was used in the FEM. The results for the ultimate load-carrying capacity obtained within the FEM are higher than those attained with Koiter’s approximation method, but the leap takes place on the identical equilibrium path as the one determined from Koiter’s theory.

## 1. Introduction

Experimental investigations [1] on the thin-walled columns made of modern materials with open cross-sections under compression have proved that the multi-mode buckling can be hazardous. When choosing the length of thin-walled elements, this effect has to be considered. These observations were confirmed in [2,3,4], where the buckling problem of laminated C-sections subject to pure bending in the flange plane [2,3] or in the web plane [4] was discussed. Attention was drawn to a rapid stability loss of beams for this type of loading. An unexpected phenomenon of stability loss of C-section beams of medium length subject to bending in the web’s plane was observed, which was interpreted as a sudden influence of the secondary global distortional buckling mode characterized by a significantly higher value of the bifurcation stress than the primary mode [5]. The phenomenon was further investigated in [6,7] to define for which lengths this phenomenon is the most dangerous. The results of the experimental investigations and the calculations conducted with a semi-analytical method based on Koiter’s asymptotic theory of thin-walled C-section beams in the web’s plane of medium length were verified with the FEM calculations [8]. A significant effect of the higher global distortional mode on the load-carrying capacity of such beams was confirmed. With steel C-section and top-hat columns subject to compression, a catastrophic unexpected influence of the secondary global distortional mode on the load-carrying capacity of the structure was indicated as well [3]. In [9], attention was paid to an effect of the distribution of inner forces, in particular, the global distortional longitudinal force, on an interaction of buckling modes and the load-carrying capacity of composite structures under bending. An influence of the secondary global mode on the load-carrying capacity of C-section beams of medium length under bending, made of a step-variable gradient material, was analyzed as well [10]. One of important conclusions from this work is such that a layer of glue connecting both layers of materials can be neglected in the modeling of such plates.

In stability of thin-walled structures, the determination of bifurcational values, which corresponds to a solution to the problem of eigenvalues, is one of fundamental issues. An eigenvector corresponds to eigenvalues. With structure stability, it is most often a buckling mode that is one of eigenvectors. It is also possible to determine inner sectional forces, including membrane forces. However, it should be remembered that eigenvalues are determined with an accuracy up to a certain constant. In [9], the key role of inner longitudinal forces corresponding to global distortional modes and of local distortional buckling modes was pointed out. These modes rarely correspond to the lowest bifurcational values of thin-walled structures [3,6,7,8,9,10,11]. An effect of distortional modes on post-buckling equilibrium paths and the load-carrying capacity of structures was also investigated in [12,13,14,15,16,17,18].

Many bifurcational values, buckling modes, and components of membrane inner forces for various numbers of buckling half-waves along the longitudinal direction (or for the assumed length of the structure) can be determined for the linear eigenproblem, which is possible thanks to, among others, the semi-analytical method (SAM) based on Koiter’s theory [6,7,8,9,11,19]. It should be mentioned that distributions of membrane inner forces are in balance. It is possible to indicate an eigenvalue and its corresponding eigenvectors, which can affect a rapid unexpected stability loss, because of the linear solution. This problem has become a starting point for the study presented here. Thin-walled hybrid columns in compression, with channel and top-hat cross-sections, were chosen as numerical examples to conduct the analysis. Column walls consist of two isotropic layers with step-variable material constants, i.e., an inner material A layer and an outer material B layer, as in [10]. Both layers are permanently integrated. In the numerical modeling, an effect of the layer connecting both the materials was not considered.

A multimodal approach to the phenomenon of stability loss of thin-walled columns in the elastic range was applied in the study. Numerical simulations were conducted with two methods, namely: the semi-analytical method (SAM) based on Koiter’s theory [19,20,21] and the finite element method (FEM), which was to verify the results obtained with the first method. In the SAM, a plate model was used to analyze individual walls of the hybrid column employing the classical laminate plate theory (CLPT). Differential equations of the plate system under analysis were formulated with a variational calculus. A nonlinear solution to the stability loss problem was attained with a perturbation method, including additionally the full Green’s strain tensor for thin-walled plates, the second Piola–Kirchhoff’s stress tensor in Lagrange’s description, and a numerical method of the transition matrix using Godunov’s orthogonalization [8,19,21].

## 2. Formulation of the Problem

**Figure 1:**
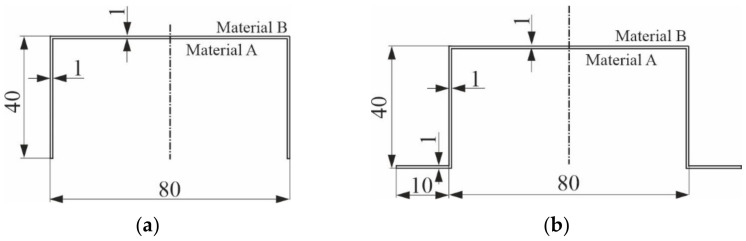
Cross-sections of the hybrid columns under analysis and their dimensions in millimeters: C-section (**a**) and TH-section (**b**).

Multimodal buckling of hybrid columns with two types of cross-sections, namely: channel (Figure 1a) and top-hat (Figure 1b), made of two isotropic materials of step-variable gradation of material properties, i.e., of material A and material B, in the elastic range and subject to compression, was considered. The cross-section does not alter along the whole length of the column. The thickness of each layer is the same and equal to 0.5 mm. The total thickness of the walls under analysis is 1 mm. The materials of the columns are made to satisfy Hooke’s law. The material constants for the material A layer are: Young’s modulus 71 GPa, Poisson’s ratio 0.33, and for the material B layer 107 GPa and 0.34, respectively. The columns under study are simple supported at both ends.

### 2.1. Semi-Analytical Method (SAM)

The nonlinear problem of stability loss in thin-walled structures within Koiter’s first-order approximation theory [19] was solved within the modified analytical-numerical method (ANM). In this approach, a precise transition matrix method is applied in contrast to the finite strip method. In the case under study, the analytical-numerical method should be additionally extended by the second-order approximation of Koiter’s theory. In the proposed solution, second-order coefficients are estimated within the semi-analytical method (SAM) [8,21], in which it is postulated that values of these coefficients are defined based on the eigenproblem. It causes the SAM approach to yield a lower-bound estimation of post-buckling equilibrium paths. The SAM allows for solving the nonlinear problem of stability of thin-walled structures made of arbitrary materials, including hybrid ones.

The structures under consideration are prismatic thin-walled columns built of plates connected along longitudinal edges. In order to account for all buckling modes, from global through local to the coupled ones, a plate model (i.e., 2D) of thin-walled structures was employed. Columns were freely supported at their both ends. Exact geometrical relationships (i.e., full Green’s strain tensor) were assumed for each plate [19,21]:

(1) $$\varepsilon_{x}=u_{,x}+\frac{1}{2}\left(w_{,x}^{2}+v_{,x}^{2}+u_{,x}^{2}\right)\;\varepsilon_{y}=v_{,y}+\frac{1}{2}\left(w_{,y}^{2}+v_{,y}^{2}+u_{,y}^{2}\right)\;2\varepsilon_{xy}=\gamma_{xy}=u_{,y}+v_{,x}+w_{,x}w_{,y}+u_{,x}u_{,y}+v_{,x}v_{,y}$$

and

(2) $$\kappa_{x}=-w_{,xx}\;\;\;\kappa_{y}=-w_{,yy}\;\;\;\kappa_{xy}=-w_{,xy}$$

where: *u*, *v, w* are displacements along the axes: *x*, *y*, *z*.

According to Koiter’s theory, the fields of displacement *U* and the fields of sectional forces *N* and *M* are distributed into a series regarding the dimensionless amplitude of deflection of the *r*-th buckling mode normalized to the thickness of the first component plate (i.e., *ζ_r_*) [19,20,21]:

(3) $$U\equiv \left(u,v,w\right)=\lambda U_{0}+\zeta_{r}U_{r}+\zeta_{r}^{2}U_{rr}+\cdots \;N\equiv \left(N_{x},N_{y},N_{xy}\right)=\lambda N_{0}+\zeta_{r}N_{r}+\zeta_{r}^{2}N_{rr}+\cdots \;M\equiv \left(M,M_{y},M_{xy}\right)=\zeta_{r}M_{r}+\zeta_{r}^{2}M_{rr}+\cdots$$

where: *λ* is dimensionless load, components of the fields of displacement, and sectional forces *N* and *M*: *U*_0_, *N*_0_ in the pre-buckling state, *U_r_*, *N_r_*, *M_r_* in the post-buckling state (first-order approximation), *U_rr_*, *N_rr_*, *M_rr_* second-order approximation. The index *r* = 1, …, *J* corresponds to the assumed number of the eigenvalue, whereas *J* is a number of eigenvalues considered in the analysis. It should be added that in the proposed formulation, a summation principle over the repeating index holds.

In thin-walled structures with the geometrical imperfections *Ū* (i.e., only linear initial geometrical imperfections corresponding to the *r*-th buckling mode are accounted for, i.e., ${\bar{U}}_{r}=\zeta_{r}^{*}U_{r}$), the total potential energy can be expressed in the following form [19,20,21]:

(4) $$\Pi =-\frac{1}{2}{\left(\frac{\sigma }{\sigma_{r}}\right)}^{2}\bar{a_{0}}+\frac{1}{2}\overset{J}{\underset{r=1}{\sum }}\bar{a_{r}}\zeta_{r}^{2}\left(1-\frac{\sigma }{\sigma_{r}}\right)+\frac{1}{3}\overset{J}{\underset{p}{\sum }}\overset{J}{\underset{q}{\sum }}\overset{J}{\underset{r}{\sum }}\bar{a_{pqr}}\zeta_{p}\zeta_{q}\zeta_{r}+\frac{1}{4}\overset{J}{\underset{r}{\sum }}\bar{b_{rrrr}}\zeta_{r}^{3}-\overset{J}{\underset{r}{\sum }}\frac{\sigma }{\sigma_{r}}\bar{a_{r}}\zeta_{r}^{*}\zeta_{r}$$

where the indices assume the values: *p* = 1, …, *J*, *q* = 1, …, *J*, *r* = 1, …, *J*. For the potential energy written in such a way (4), a system of equilibrium equations can be written shortly as:

(5) $$\left(1-\frac{\sigma }{\sigma_{r}}\right)\zeta_{r}+a_{pqr}\zeta_{p}\zeta_{q}+b_{rrrr}\zeta_{r}^{3}=\frac{\sigma }{\sigma_{r}}\zeta_{r}^{*}$$

where:

(6) $$a_{pqr}=\bar{a_{pqr}}/\bar{a_{r}}$$

(7) $$b_{rrrr}=\bar{b_{rrrr}}/\bar{a_{r}}$$

are the coefficients of the first-order (6) and second-order (7) nonlinear approximation, correspondingly [19,20,21]. The eigenvalues *U_r_* are mutually orthogonal, which can be expressed as: σ_0_ *l*_11_(*U_I_*,*U_K_*) = 0, (*I*,*K*) = [1,*J*], *I* ≠ *K*, where *J* is a number of all buckling modes that are considered essential in the structure performance and were subject to the analysis. It allows the coefficients occurring in Formula (6) to be written in the form:

(8) $$\bar{a_{r}}=-\lambda_{r}\sigma_{o}\cdot l_{2}\left(U_{r}\right)$$

(9) $$\bar{a_{pqr}}=\sigma_{p}\cdot l_{11}\left(U_{q},U_{r}\right)+0.5\sigma_{r}\cdot l_{11}\left(U_{p},U_{q}\right)$$

The above coefficients (Equations (6), (8) and (9)) depend only on eigenvalues. This significantly simplifies solving the nonlinear problem of stability loss in the first-order approximation. The first term in relationship (9) is described with the formula:

(10) $$\begin{array}{cc} \sigma_{p}\cdot l_{\mathrm{11}}(U_{q},U_{r})= & \sum_{i=1}^{n}\iint [N_{xp}\left(u_{q,x}u_{r,x}+v_{q,x}v_{r,x}+w_{q,x}w_{r,x}\right)+N_{yp}\left(u_{q,y}u_{r,y}+v_{q,y}v_{r,y}+w_{q,y}w_{r,y}\right)\\ & +N_{xyp}\left(u_{q,x}u_{r,y}+u_{q,y}u_{r,x}+v_{q,x}v_{r,y}+v_{q,y}v_{r,x}+w_{q,x}w_{r,y}+w_{q,y}w_{r,x}\right)]dxdy \end{array}$$

where *n*—number of component plates that form the column under analysis.

It follows from relationship (10) that the sectional forces corresponding to the eigenvalue denoted with the index p (i.e., $N_{xp},N_{yp},N_{xyp}$) exert a considerable influence on magnitudes of the three-index coefficients ${\bar{a}}_{pqr}$ (Equation (9)). Because with these indices integrals of different signs are summed up, it should be remembered that the symmetrical modes regarding the axis of the cross-section can interact with an arbitrary number of these modes, whereas the anti-symmetrical modes interact only with anti-symmetrical pairs and with an arbitrary number of the symmetrical ones. With columns freely supported, solutions along the column length are developed into a sinus function, which causes subsequent eigenmodes to differ in the number of half-waves (further denoted as m) that appear along the column length. In the case of columns that are medium-long and long, global buckling occurs when one half-wave occurs, i.e., *m* = 1, and local buckling when the number of these half-waves is higher, i.e., m ≠ 1. To each buckling mode, for instance, denoted as *r*, a number of half-waves *m_r_* can be assigned. It follows from the conducted simulations that the three-index coefficients $a_{pqr}$ (Equation (6)) can be neglected when the number of half-waves differs considerably, i.e., $\left|m_{q}-m_{r}\right|>>0$ for the selected eigenvalues. Choosing the eigenvalues for analysis, one should follow the principle that these modes have an approximate number of half-waves that occurred along the column length. It has been found that these coefficients (Equation (6)) are equal to 0, when taking three eigenvalues to the analysis, a relationship holds that such that a sum of the numbers of their half-waves (i.e., *m_p_* + *m_q_* + *m_r_*) is an even number. Such an approach allows one to determine precisely the values of the coefficients *a_pqr_*, according to the applied Byskov and Hutchinson’s nonlinear theory [19,21]. The one-index coefficients ${\bar{a}}_{r}$ (Equation (8)) depend on fractional values. Attention should be paid to the fact the coefficients *a_pqr_* according to (6) introduced to equilibrium Equation (5) refer to subsequent values of bifurcational loads, whereas the coefficients ${\bar{a}}_{pqr}$ are independent and determined from Formula (9). The condition of zeroing of the Jacobian of system of Equation (5) corresponds to the boundary point (i.e., the ultimate load-carrying capacity or the maximal load) or to the point of secondary bifurcation.

The dimensionless shortening of the column ends Δ/Δ_min_ was determined as a function of the dimensional stress σ/σ_min_ by differentiation of the expression for potential energy (4) with respect to σ/σ_min_ [8,19,21]

(11) $$\frac{\Delta }{\Delta_{min}}=\frac{\sigma }{\sigma_{min}}\left[1+\frac{\sigma_{min}}{\sigma \bar{a_{0}}}\overset{J}{\underset{r=1}{\sum }}\frac{\sigma }{\sigma_{min}}\bar{a_{r}}\zeta_{r}\left(0.5\zeta_{r}+\zeta_{r}^{*}\right)\right]$$

where: Δ_min_—minimal shortening of the column corresponding to the minimal value of the bifurcational load σ_min_.

### 2.2. Finite Element Method (FEM)

**Figure 2:**
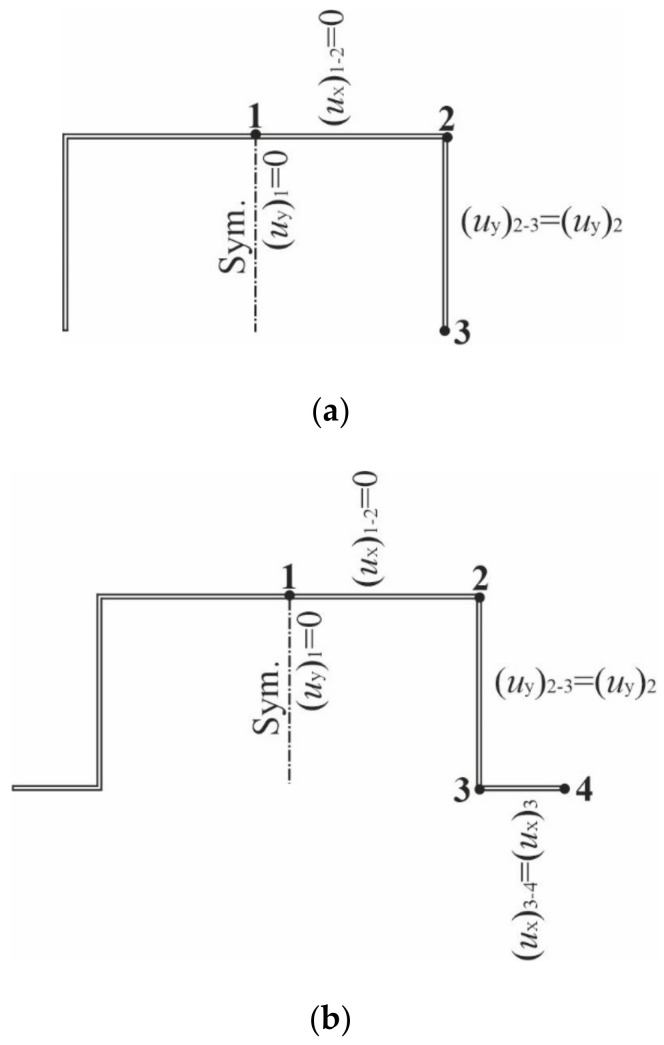
Boundary conditions for the C-channel (**a**) and the TH-channel (**b**) assumed in the finite element method (FEM) simulations [3].

The verifying simulations were conducted with the finite element method (FEM) employing a commercial package Abaqus [22]. A numerical model of the thin-walled structures under analysis was generated with four-node shell elements of eight degrees of freedom in each node (element type: S8R—eight-node doubly curved shell, reduced integration). The element size was determined through the convergence analysis to be equal to 2 mm. Boundary conditions (Figure 2) were adapted to the requirements imposed by the SAM [23,24]:

1. In the center of the column, the displacement of the whole cross-section along the column length is equal to 0, i.e., *u*_z_ = 0;
2. At both ends, it is assumed that the point of intersection of the web with the axis of symmetry of the cross-section (denoted as point 1 in Figure 2a,b) cannot displace along the *y*-axis, i.e., (*u*_y_)_1_ = 0. The web in segment 1–2 cannot displace along the *x*-axis, i.e., (*u*_x_)_1-2_ = 0. The arm in segment 2–3 displaces with respect to the *y*-axis in the same way as corner 2, i.e., (*u*_y_)_2-3_ = (*u*_y_)_2_. Reinforcement 3–4 displaces along the *x*-axis identical to corner 3 (Figure 2b), i.e., (*u*_x_)_3-4_ = (*u*_x_)_3_.

Another variant of boundary conditions, in compliance with the SAM requirements, was proposed by Szymczak and Kujawa [25,26,27,28]. Comparing the results of simulations of bifurcational loads and post-buckling behaviors for both variants of the boundary conditions, one could state that they agreed. During the verifying calculations, it was found that the determined eigenmodes and post-buckling equilibrium paths were identical in both the cases of the modified boundary conditions. It should be added that the nonlinear stability problem was solved both with the Riks algorithm and the Newton–Raphson algorithm. The results did not differ, thus the present work shows solely the results for the Riks algorithm, which allows for tracing not only stable equilibrium paths. To obtain the numerical convergence and a possibility to estimate the maximal load, the time-step was very fine (i.e., total time-step was 1, initial: 0.001, maximum: 0.01, and minimum: 1 × 10^−20^). The SAM is much more efficient and allows for a much simpler analysis of the phenomena under investigation and their interpretation when compared to the FEM. Its main limitation lies in a necessity to select properly the eigenvalues, which affect considerably the post-buckling behavior of thin-walled structures. The second limitation of this method in comparison to the FEM is the fact that several eigenmodes are considered in the analysis (in general, fewer than 10).

## 3. Results and Discussion

### 3.1. Channel Column

In the first stage of the numerical simulations, it was assumed for the considered hybrid (i.e., materials A–B) channel of the geometrical dimensions of the cross-section shown in Figure 1a that the outer material B layer and the inner material A layer had the identical thickness of 0.5 mm each and, for the assumed material constants, the values of the bifurcational loads (i.e., eigenvalues) were determined in a broad range of variability of the column length from 25 up to 2000 mm, assuming that only one buckling half-wave (i.e., *m* = 1) formed along its length.

**Figure 3:**
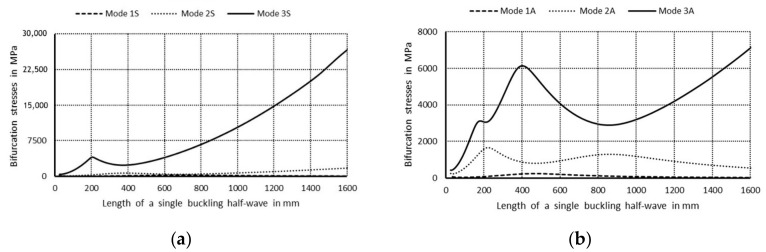
Values of the bifurcational stress in MPa versus the length of a single buckling half-wave in mm: (**a**) first three symmetrical buckling modes, (**b**) first three anti-symmetrical buckling modes.

In Figure 3a, values of bifurcational stresses are given in MPa for the first three symmetrical buckling modes, whereas in Figure 3b the first three anti-symmetrical buckling modes as a function of the length of a single buckling half-wave are expressed in mm, correspondingly. The following numerical notations of the curves were assumed: 1. Primary modes corresponding to lowest bifurcational values when m = 1; 2. Secondary modes when m = 1; 3. Third modes, higher when m = 1, and the letter notations were used: S—denotes symmetrical modes with respect to the symmetry axis of the column cross-section, A—stands for anti-symmetrical modes.

**Figure 4:**
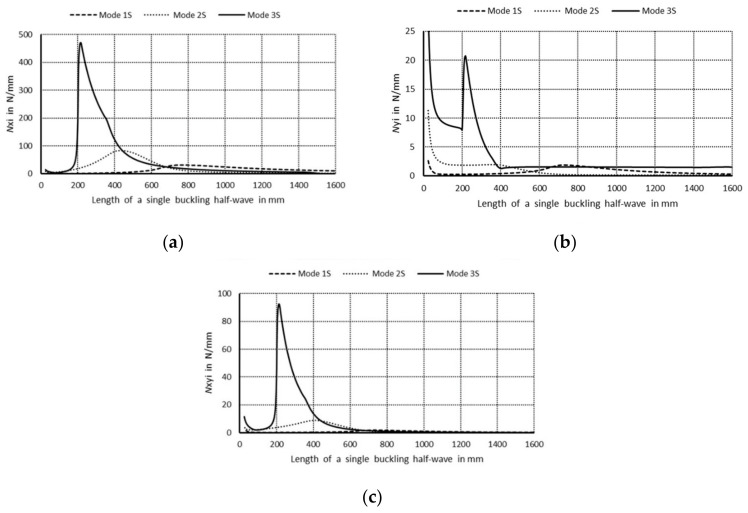
Maximal absolute values of membrane forces in N/mm for symmetrical buckling modes: (**a**) longitudinal forces *N_xi_*, (**b**) transverse forces *N_yi_*, and (**c**) shear forces *N_xyi_* as a function of the length of a single buckling half-wave in mm.

**Figure 5:**
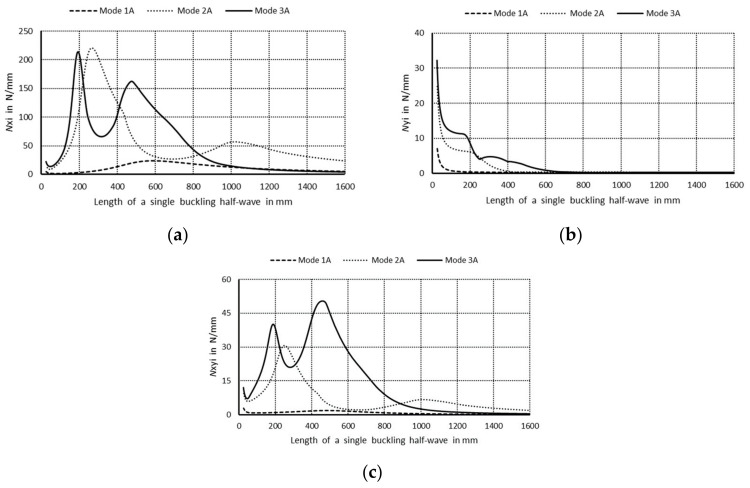
Maximal absolute values of membrane forces in N/mm for anti-symmetrical buckling modes: (**a**) longitudinal forces *N_xi_*, (**b**) transverse forces *N_yi_*, and (**c**) shear forces *N_xyi_* as a function of the length of a single buckling half-wave in mm.

The curve 1S in Figure 3a corresponds to the lowest symmetrical buckling modes when *m* = 1. The curve assumes the local minimum for the length of a single half-wave equal to 110 mm. For shorter lengths, it grows rapidly and reaches the local maximum when its length is 670 mm, then the curve descends gently for higher values of the length. The curve 2S has two local minima for the half-wave lengths: 70 and 670 mm, and it has one local maximum for the length of 380 mm. For the half-wave shorter than 1000 mm, the bifurcational stresses are lower than 1000 MPa. The local minimum for the curve 2S and the local maximum for the curve 1S occur for the same value of the half-wave equal to 380 mm. For the curve 3S, the local minimum occurs when the half-wave length is 390 mm, and the local maximum for the length of 210 mm. Bifurcational values for the curve 3S are one order of magnitude higher than for the curve 1S. In Figure 3b, the curve 1A (anti-symmetrical buckling mode) attains the local minimum for the half-wave length equal to 85 mm, the maximum for the length of 450 mm, then it descends for longer columns. The curve 2A has two local minima: for the half-wave lengths of 45 and 450 mm and two maxima for the lengths: 230 and 900 mm. In the half-wave’s range length from 150 to 1100 mm, bifurcational values of the curve 2A do not exceed 1000 MPa. Bifurcational values for the curve 3A, within the whole range of length alternations, are in practice higher than 1000 MPa, and only for the length shorter than 100 mm are inconsiderably lower. Components of membrane forces of the first-order approximation were normalized by the normalization condition of the deflection amplitude of the *i*-th buckling mode for the first component plate thickness (i.e., *ζ_i_*). In the further part of the study, according to (3b), only maximal absolute values of membrane components of the inner forces *N_xi_*, *N_yi_*, *N_xyi_*, expressed in N/mm with accuracy up to the dimensionless amplitude of the *i*-th buckling mode *ζ_i_*, will be considered. In Figure 4, maximal absolute values of membrane sectional forces are presented for symmetrical buckling modes: longitudinal forces *N_xi_* (Figure 4a), transverse forces *N_yi_* (Figure 4b), and shear forces *N_xyi_* (Figure 4c), as a function of the length of a single buckling half-wave. In Figure 5, there are analogous plots of forces for anti-symmetrical buckling modes.

As regards the plots of sectional forces (Figure 4 and Figure 5), their maximal values and the corresponding lengths of buckling half-waves along the column lengths, in particular the longitudinal components *N_xi_*, are of interest in practice as most significant in their interaction [9]. In Figure 4a, for the symmetrical modes, it is easy to see the dominant value of *N_x_*_3_ ≈ 470 N/mm when the length is equal to 220 mm—the curve 3S (i.e., when the index *i* = 3). The remaining maximal values of *N_xi_* for the curves 1S (*i* = 1) and 2S (*i* = 2) are over five times lower. For the curve 3S, the force *N*_*x*3_ is higher than 50 N/mm in the length range from 190 to 500 mm. For the curve 1S, the values of *N_x_*_1_ < 30 N/mm, whereas for 2S—*N_x_*_2_ < 90 N/mm. In Figure 4b, maximal values of the forces *N_yi_* are highest for very short channels for the length shorter than 30 mm, and they do not exceed the value of 10 N/mm for the first (*i* = 1), 15 N/mm for the second (*i* = 2), and 30 N/mm for the last buckling mode (*i* = 3), correspondingly. In Figure 4c, the maximal value *N_xyi_* occurs for the last buckling mode (*i* = 3), i.e., for *N_xy_*_3_, and it is equal to 90 N/mm for the length of 220 mm. Maximal values of *N_xy_*_1_ are lower than 5 N/mm, and *N_xy_*_3_—10 N/mm. It can be easily noticed that *N_x_*_3_ ≈ 470 N/mm (Figure 4a) and *N_xy_*_3_ ≈ 90 N/mm (Figure 4c) for the length 220 mm and, correspondingly, *N_xy_*_3_ and *N_y_*_3_ for the same length attain extreme values (Figure 4b).

In Figure 5a, for anti-symmetrical modes, the maximal values of *N_xi_* for the curves are: 1A—*N_x_*_1_ < 25 N/mm, 2A—*N_x_*_2_ < 220 N/mm, and 3A—*N_x_*_3_ < 210 N/mm, correspondingly. The values of *N_x_*_2_ and *N_x_*_3_ are close to each other and twice as low as 3S. The plots of the curves 2A and 3A are by far more complex than for symmetrical modes. The curve 1A attains its maximum for the half-wave length of 600 mm. The curve 2A has its maximum for *N_x_*_2_ ≈ 220 N/mm and the half-wave length of 270 mm and the local maximum for 60 N/mm and 1000 mm. It attains also two minima for the half-wave lengths: 50 and 670 mm. The curve 3A has two maxima for: *N_x_*_3_ = 210 N/mm and the length 200 mm and *N_x_*_3_ = 160 N/mm and the length of 470 mm, correspondingly. Its minima occur for *N_x_*_3_ = 65 N/mm and the length 315 mm, and *N_x_*_3_ = 15 N/mm and the length 50 mm. As can be easily noticed in Figure 5b, maximal values of the forces *N_y_*_2_ are equal to for 1A—*N_y_*_1_ < 10 N/mm, 2A—*N_y_*_2_ < 25 N/mm, 3A—*N_y_*_3_ < 35 N/mm. In Figure 5c, the maximal values of *N_xy_*_1_ for the curve 1A are less than 3. The curve 2A has two maxima: *N_xy_*_2_ = 30 N/mm and the length is 250 mm, and *N_xy_*_2_ = 7 N/mm and the length is 1000 mm. Its minima occur when *N_xy_*_2_ = 6 N/mm and the length is 50 mm, and *N_xy_*_2_ = 2 N/mm and the length is 660 mm. By analogy, for the curve 3A, we have the maxima for: *N_xy_*_3_ = 40N/mm and the length of 200 mm, and *N_xy_*_3_ = 50 N/mm and the length 475 mm, and the minima for: *N_xy_*_3_ = 21 N/mm and the length 285 mm, and *N_xy_*_3_ = 7 N/mm and the length 50 mm. The maximal values of *N_x_*_2_ and *N_x_*_3_ (curves 2A and 3A in Figure 5a) occur for the same values as for *N_x_*_2_ and *N_x_*_3_ (curves 2A and 3A in Figure 5c).

The next stage consisted in a nonlinear analysis of interactive buckling, which allowed for determination of post-buckling equilibrium paths and, possibly, the load-carrying capacity (i.e., maximal loading) of the hybrid channel. Two total column lengths, for which absolute maximal values of the longitudinal forces *N_xi_* were the highest (cf. Figure 4a and Figure 5a), were selected for the analysis to observe strong interactions between the chosen eigenmodes. The following total lengths (further denoted as *L*) of the channel were assumed in the analysis: 210 and 250 mm. Table 1 lists values of bifurcational stresses for two assumed total lengths of the channel determined with two methods, namely: the SAM and the FEM. For the SAM, maximal absolute values of membrane sectional forces and several half-waves (in brackets) along the longitudinal direction that form along the length of the columns under analysis (denoted as the parameter *m*) and letter notations of the conditions on the axis of symmetry of the cross-section (i.e., S or A), are given. It should be added that for each variant denoted with the parameter m and the letter S or A, there are many eigenmodes, which are described: the lowest one is referred to as the primary eigenmode, whereas the subsequent ones, which are higher eigenmodes that differ from the primary one by deformations of the cross-section, can be found. The values of local bifurcational stresses are given for the buckling mode of two half-waves along the length (i.e., *m* = 2), as for this number of half-waves, the value of the bifurcational load is the lowest as well as for the mode of a single half-wave along the length (i.e., *m* = 1). It follows from a comparison of bifurcational loads that the SAM and FEM results are in high conformity. The maximal error for all the cases does not exceed 3%.

**Figure 6:**
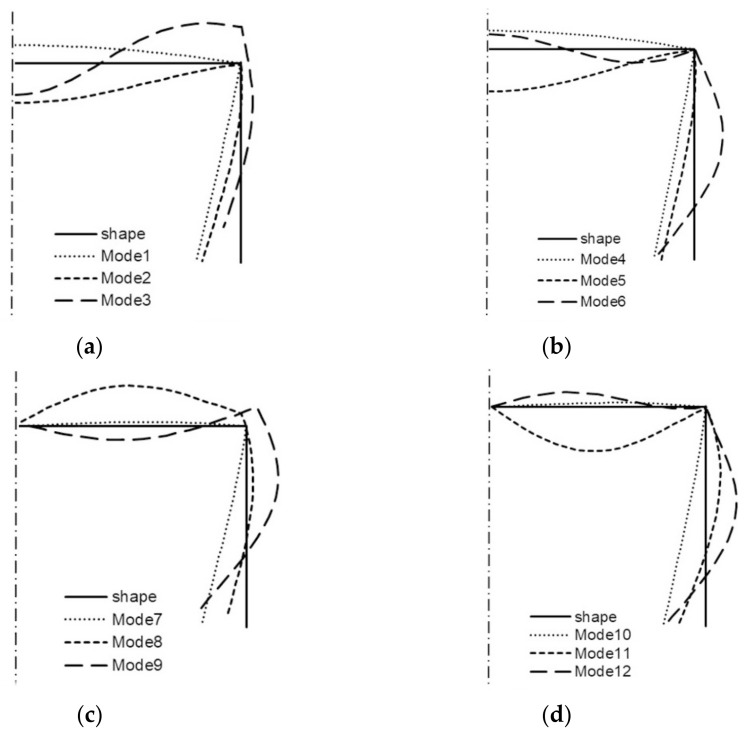
Buckling modes of the channel of the total length *L* = 210 mm (Table 1): symmetrical modes 1, 2, 3 (**a**), modes 4, 5, 6 (**b**), and anti-symmetrical modes 7, 8, 9 (**c**), modes 10, 11, 12 (**d**).

**Figure 7:**
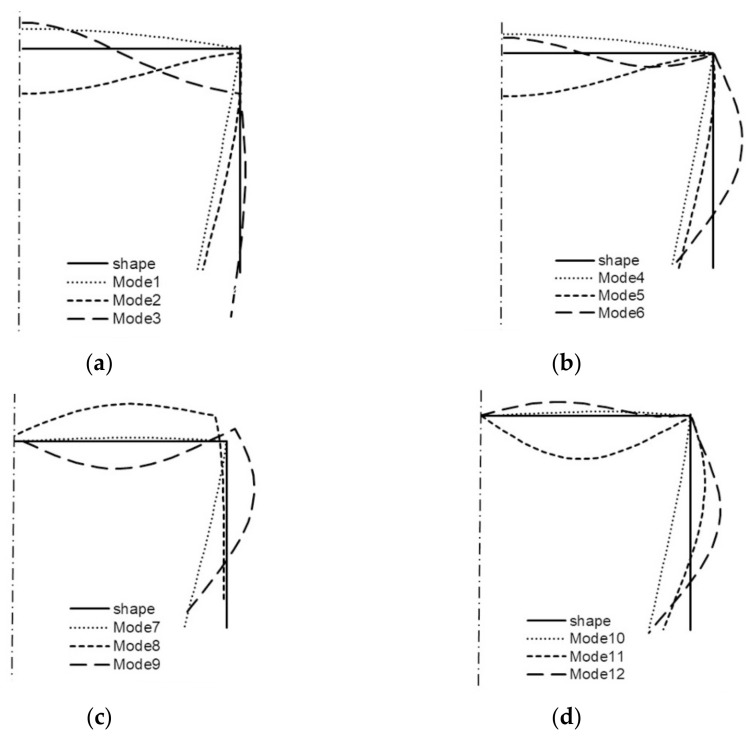
Buckling modes of the channel of the total length *L* = 250 mm (Table 1): symmetrical modes 1, 2, 3 (**a**), modes 4, 5, 6 (**b**), and anti-symmetrical modes 7, 8, 9 (**c**), modes 10, 11, 12 (**d**).

All the buckling modes from Table 1 for two lengths of the channel are shown in the next figures (Figure 6 and Figure 7). For *L* = 210 mm, it is shown in Figure 6 that:

(a) modes 1–3 are symmetrical modes, corresponding to subsequent modes with one half-wave along the column length (i.e., *m* = 1);
(b) modes 4–6 are symmetrical modes, corresponding to subsequent modes with two half-waves along the column length (i.e., *m* = 2);
(c) modes 7–9 are anti-symmetrical modes, corresponding to subsequent modes with one half-wave along the column length (i.e., *m* = 1);
(d) modes 10–12 are anti-symmetrical modes, corresponding to subsequent modes with two half-waves along the column length (i.e., *m* = 2).

By analogy, all the modes from Table 1 when *L* = 250 mm are shown in Figure 7. Comparing the buckling modes for the total length 210 mm (Figure 6), it can be easily noticed that for nearly all 12 buckling modes, the channel corner does not displace except for the symmetrical mode—mode 3—when one half-wave occurs along the column length (i.e., *i* = 3 and *m* = 1S) and for two anti-symmetrical modes—modes 8, 9—also, when one half-wave occurs along the column length (i.e., *i* = 8–9 and *m* = 1A). For these modes, the corners displace, and this is caused by an appearance of large longitudinal membrane forces *N_xi_* when *i* = 3, 8, 9. These modes correspond to distortional buckling modes. Identical conclusions follow for the channel of the length *L* = 250 mm (Figure 7). Comparing the results for both the lengths and the maximal absolute values of the forces *N_xi_* for buckling modes 3, 8, 9 (Table 1) to the corresponding buckling modes, one can see that there is a coupling between them, that is to say, on the basis of the displacement of the corner for the given mode, it can be stated that it is caused by a considerable inner longitudinal force. The reverse reasoning is correct as well.

In the SAM, it is possible for the nonlinear problem to consider a strictly defined and finite number of buckling modes within an interaction of these modes. This allows for determining crucial modes that decide post-buckling equilibrium paths and the load-carrying capacity of the structure. When an interaction of *J* modes is considered, it means that the numerical model has such a number of degrees of freedom. In the FEM, it is not possible to determine which modes are to be considered in the numerical model. Both in the SAM and the FEM, a certain number of modes with inaccuracies and their amplitude should be assumed to solve a nonlinear problem. In this study, it was assumed for the SAM that the amplitude of the initial deflection was equal to: |ζ_r_^*^| = 0.2 (Formula (5)), where *r* = 1, 2, …, *J*. The imperfection signs were chosen in the most dangerous way, i.e., to obtain the lowest value of the ultimate load-carrying capacity or the least slope equilibrium path. In the FEM, identical values of imperfections were assumed of course and their signs were chosen in the same way.

In Table 2, the dimensionless ultimate load-carrying capacities σ_S_/σ_4_ obtained with the SAM and the FEM for both lengths of channels under consideration are presented. In the SAM, 3-, 4- and 6-modal approaches were considered, selecting various combinations of modes (Table 1—buckling modes 1–12). For the first two, only symmetrical modes were considered, whereas when anti-symmetrical modes were also accounted for, a 6-modal approach was applied. This was caused by the necessity to consider an even number of anti-symmetrical modes in the interaction. Indices of buckling modes (i.e., *i*-index), which were considered in the SAM, and the dimensionless value of the ultimate load-carrying capacity referring to the minimal value of the buckling load, i.e., σ_s_/σ_min_ = σ_s_/σ_4_ for the given total length of the column, were also given. In the case of the channel length *L* = 210 mm, for an interaction of three modes, the ultimate load-carrying capacity was not obtained. The post-buckling equilibrium path was an ascending curve.

In a 4-modal approach, the dimensionless ultimate load-carrying capacity σ_s_/σ_4_ is equal to 1.42 for an interaction of symmetrical modes. An increase in the number of modes up to six in the first case, i.e., when only anti-symmetrical modes with one half-wave along the length (*m* = 1) are accounted for, does not result in a change in the load-carrying capacity when compared to the 4-modal approach. When interactions of mode 10 (*m* = 2A) and modes 8 or 9 (*m* = 1A) are considered in the 6-modal approach, a significant lowering of the load-carrying capacity σ_s_/σ_4_ ≈ 0.94 takes place. For modes 8 and 9 we have much higher values of longitudinal forces than for mode 7. In the FEM, 4 and 6 different imperfections corresponding to the modes of initial deflections considered in the SAM were assumed, and the lowest value of the dimensionless load-carrying capacity σ_s_/σ_4_ equaled 1.49. In the FEM, it was not possible to confirm the SAM results obtained when anti-symmetrical modes were taken into consideration. The FEM load-carrying capacity corresponds to the SAM 4-modal approach. Similar differences in the evaluation of the load-carrying capacity with a coupled interaction of symmetrical modes in the SAM and an invisible effect of these modes on the load-carrying capacity in the FEM were found in [3].

**Figure 8:**
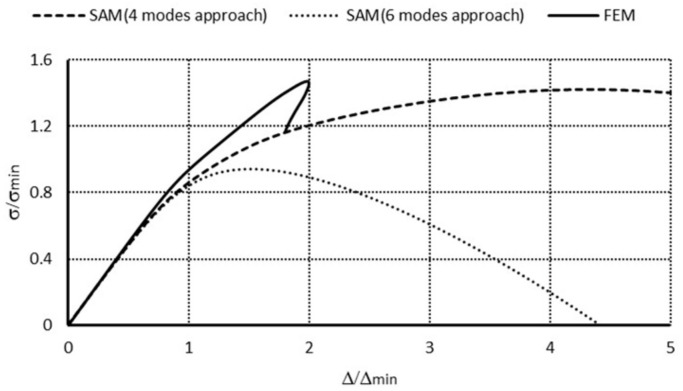
Comparison of dimensionless equilibrium paths for the channel of total length *L* = 210 mm.

**Figure 9:**
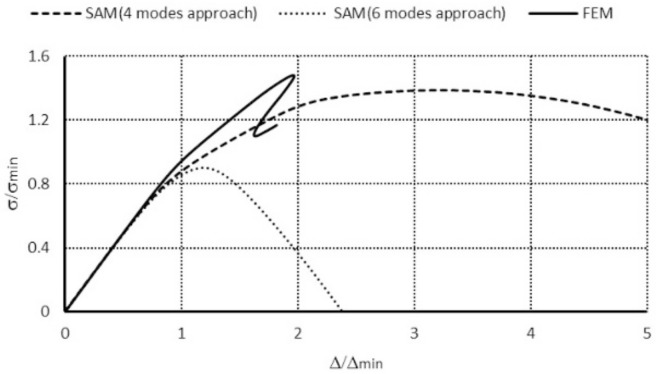
Comparison of dimensionless equilibrium paths for channels of the total length *L* = 250 mm.

For the channel of the total length *L* = 250 mm for each modal approach, the ultimate load-carrying capacity (i.e., σ_S_/σ_4_) was attained. However, in a 3-modal approach, the load-carrying capacities differed 1.4 times from the other approaches. In a 4-modal approach for symmetrical modes, σ_s_/σ_4_ = 1.39 was obtained similar to the 6-modal approach, in which two anti-symmetrical modes (modes 8, 9 when *m* = 1A, Table 1) are accounted for. For the 6-modal approach, a lower value of the load-carrying capacity was obtained at anti-symmetrical modes 10 (*m* = 2A) and 8 (*m* = 1A) than for modes 10 and 9. This difference was equal to 1.15 times. It results from the fact that *N_x_*_8_>*N_x_*_9_ and σ_8_ < σ_9_. From the FEM, σ_s_/σ_4_ = 1.49 was obtained. Similarly, as for the length of *L* = 210 mm, an effect of the anti-symmetrical modes on the load-carrying capacity could not be confirmed in the FEM. The load-carrying capacities for two different column lengths from the FEM are identical. It should be remembered that the SAM yields a lower-bound evaluation of the load-carrying capacity and foresees the ultimate load-carrying capacity with accuracy below 10% regarding the FEM for symmetrical modes. Because of the time involved in numerical calculations, this outcome is very satisfactory. Considering the results from [3], the authors think that further investigations on an influence of anti-symmetrical modes on interactive buckling and load-carrying capacity should be carried out. In Figure 8, post-buckling equilibrium paths in the system: dimensionless compressive stress–dimensionless column shortening (i.e., (σ_s_/σ_min_) − (Δ_s_/Δ_min_), where σ_min_ corresponds to the lowest buckling stress, whereas Δ_min_—denotes the shortening corresponding to this stress) obtained within the SAM and the FEM for the channel of the total length *L* = 210 mm are presented. An analogous plot for the length *L* = 250 mm is drawn in Figure 9.

In Figure 8, the curve representing the SAM 4-modal approach reaches its maximal value for the dimensionless shortening equal to 4.5, whereas for the FEM it is 2.0 at approximate values of the dimensionless ultimate load-carrying capacity σ_s_/σ_min_. The FEM model is more rigid than the SAM model. It should be remembered that in the SAM only up to six degrees of freedom were considered, whereas the FEM model had four orders of magnitude more. A nonlinear analysis was conducted only for the elastic range. The dimensionless ultimate load-carrying capacity attained with the SAM equaled 0.9, and for the FEM, after a jump on the identical path as in the SAM simulations, it was approx. 1.2 at the point the calculations were disrupted by a loss of convergence. For the SAM 6-modal approach, according to the conclusions from [3], numerical calculations should be continued from the authors’ viewpoint.

Similar conclusions can be drawn for the channel of the length equal to *L* = 250 mm (Figure 9). In the case of the FEM simulations, the ultimate value occurs when the dimensionless shortening is 2.0. Thus, a slight alternation in the length (about 1.2 times) causes the influence of global modes to be much more considerable, as the lowest bifurcational values for local modes are practically the same for both lengths. The FEM post-buckling path falls down after it reaches the value of the ultimate load-carrying capacity and the curve clearly reaches the minimum for σ/σ_4_ ≈ 1.2, and then the FEM code disrupts the calculations. In the authors’ opinion, new buckling modes appear at the points the FEM calculations are disrupted (i.e., at σ/σ_4_ ≈ 1.1) for both lengths of the channels under analysis. This causes the SAM and FEM results to come closer. They should be subject to further in-depth analysis.

**Figure 10:**
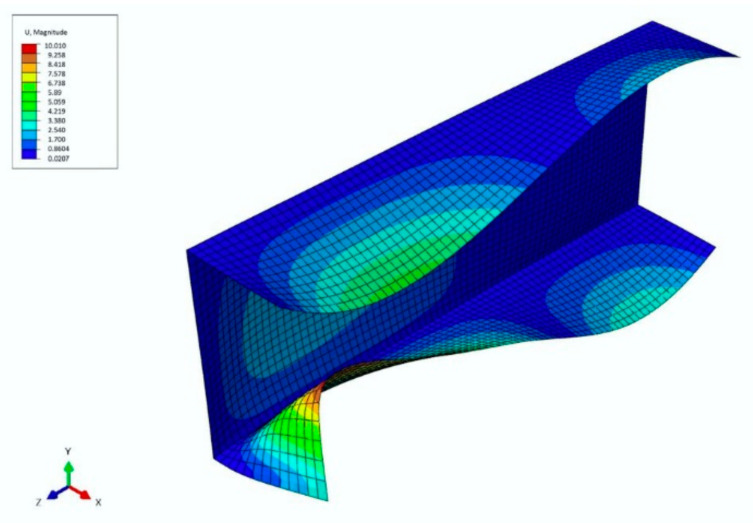
Collapse mode for the channel of lengths *L* = 210 mm/250 mm in the ultimate point.

The next figure shows a collapse mode that corresponds to the ultimate load-carrying capacity σ_s_/σ_4_ of channels of the lengths: *L* = 210 mm and *L* = 250 mm (Figure 10). The FEM collapse mode for both the column lengths are identical. Two buckling half-waves appear along the channel length. In the half-length, a visible deviation from the straight line can be seen, which corresponds to an effect of the distortional buckling mode.

### 3.2. Top-Hat Column

The second thin-walled column subject to consideration is a hybrid column with a top-hat cross-section of the geometrical dimensions of the cross-section shown in Figure 1b. An addition of external boundary reinforcements causes stiffening of the structure compared to the channel and thus, enlarges bifurcational loads. Identically as in the channel previously discussed, it is assumed that the outer layer is made of material B, and the inner layer is made of material A. The thicknesses and the material constants are the same as in the previously discussed column.

**Figure 11:**
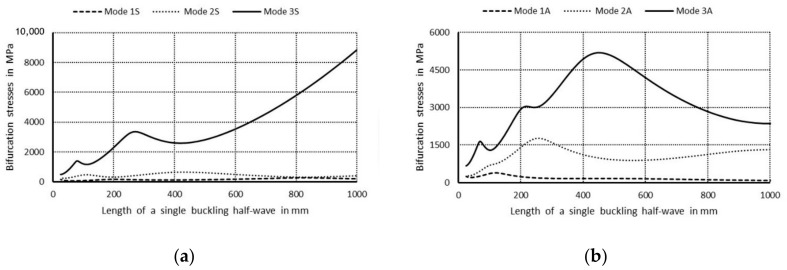
Values of bifurcational stress in MPa as a function of the length of one buckling half-wave in mm: (**a**) first three symmetrical buckling modes, (**b**) first three anti-symmetrical buckling modes.

In Figure 11, a variability in bifurcational loads as a function of the length of one buckling half-wave for symmetrical modes (Figure 11a) and for anti-symmetrical modes (Figure 11b) between lengths from 25 to 1000 mm, which is most interesting from the practical point of view, is shown. Like in Figure 3, the following notations of curves are assumed: 1—primary mode, 2—secondary mode, 3—third mode, S—symmetrical mode, A—anti-symmetrical mode. The curve 1S in Figure 11a attains two maxima for the lengths of buckling half-wave equal to approx. 200 and 800 mm and two minima for the lengths of approx. 70 and 400 mm. The curve 2S has maxima for half-wave lengths of approx. 80 and 425 mm, and minima for 40 and 200 mm. The curve 3S attains maxima, correspondingly, for 90 and 275 mm, and minima for 25 and 120 mm. For the range of variability in length under analysis, the maximal bifurcational stresses for the curves: 1S are lower than 315 MPa, 2S: 650 MPa, 3S: 9000 MPa. It can also be noticed that when the half-wave lengths are 200 and 825 mm, the curve 1S reaches maxima, and the curve 2S minima. For the anti-symmetrical modes (Figure 11b), the curve 1A has one minimum for the length of about 40 mm and one maximum for 120 mm, then it descends for higher lengths. The curve 2A has two maxima for the half-wave lengths of about 250 and 1000 mm, and two maxima for 35 and 550 mm. The curve 3A has three local maxima for 75, 220, and 450 mm, and three minima for 100, 250, and 1000 mm. The maximal values of bifurcational loads for individual curves are not higher than: 1A: 400 MPa, 2A: 1800 MPa, 3A: 5200 MPa.

**Figure 12:**
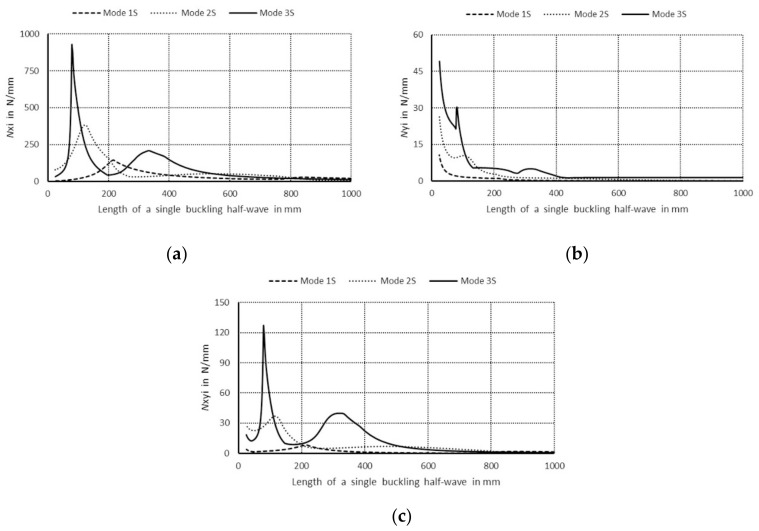
Maximal absolute values of membrane forces in N/mm for symmetrical buckling modes: (**a**) longitudinal forces *N_xi_*, (**b**) transverse forces *N_yi_* and (**c**) shear forces *N_xyi_* as a function of the length of a single buckling half-wave in mm.

**Figure 13:**
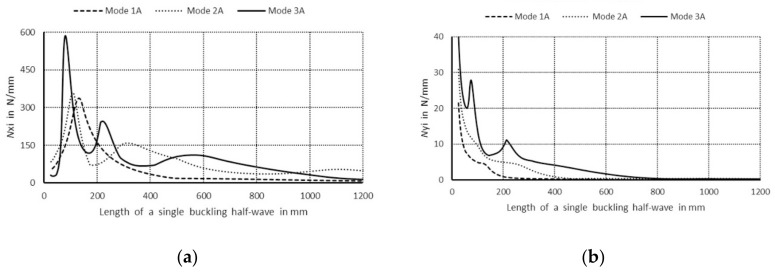

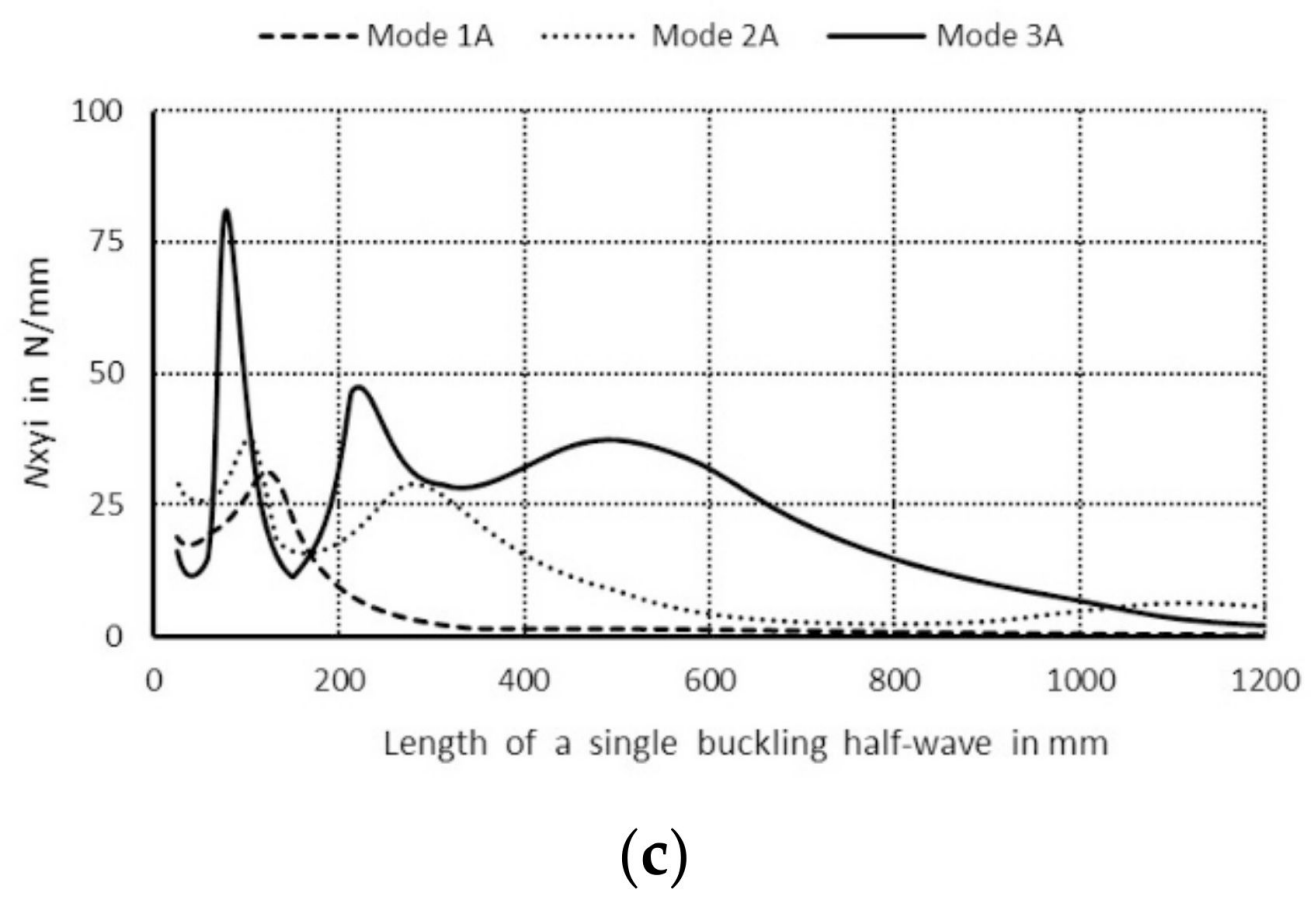
Maximal absolute values of membrane forces in N/mm for anti-symmetrical buckling modes: (**a**) longitudinal forces *N_xi_*, (**b**) transverse forces *N_yi_* and (**c**) shear forces *N_xyi_* as a function of the length of a single buckling half-wave in mm.

Next, in Figure 12 and Figure 13, maximal absolute values of membrane sectional forces for symmetrical and anti-symmetrical buckling modes, respectively, are presented. As discussed in Section 3.1 for hybrid channels, the most important are maximal values of the longitudinal forces *N_xi_* when *i* = 1,2,3. For the first symmetrical mode of the longitudinal force (curve 1S in Figure 12a), the maximal value is attained when the length of a single buckling half-wave is equal to 220 mm, and it exceeds the value of 100 N/mm when the half-wave length changes from 185 to 250 mm. Respectively, the curve 2S reaches its maximum when the half-wave length is 125 mm and exceeds 100 N/mm in the range from 60 to 225 mm, whereas the curve 3S attains the maximum when the half-wave lengths equal 80 and 330 mm and exceeds the value of 100 N/mm in the ranges: from 60 to 160 mm and from 260 to 440 mm. Membrane transverse forces (Figure 12b) attain maximal values for the curves: 1S: 15 N/mm, 2S: 30 N/mm, and 3S: 45 N/mm. Membrane shear forces are (Figure 12c), correspondingly: 1S: 10 N/mm, 2S: 40 N/mm, and 3S: 124 N/mm. The values for 3S above 100 N/mm are in a narrow range of variability for the length of about 80 mm.

Maximal values of cross-sectional forces for anti-symmetrical modes are presented in Figure 13. Maximal longitudinal forces are (Figure 13a), correspondingly, for the curves: 1A has a maximal value of 300 N/mm and for lengths 60–250 mm are higher than 100 N/mm; the curve 2A has two maxima: 350 N/mm for the range 50–170 mm and 160 N/mm for 250–480 mm; the curve 3A has two maxima as well: 600 and 200 N/mm for the range 60–300 mm. As can be easily noticed in Figure 13b, transverse forces do not exceed the following values for the curves: 1A: 25 N/mm, 2A: 35 N/mm, 3A: 45 N/mm. Shear forces are (Figure 13c), correspondingly: 1A: 30 N/mm, 2A: 40 N/mm, 3A: 80 N/mm. Comparing the plots for TH-columns (Figure 12 and Figure 13) to C-columns (Figure 4 and Figure 5), maximal values of inner forces occur for shorter lengths of structures under compression, which is caused by boundary reinforcements of TH-columns in relation to C-columns.

Considering maximal absolute values of longitudinal forces in evaluation of their influence on the load-carrying capacity of hybrid TH-channels, the following lengths were selected: 80, 130, 225, and 330 mm. In Table 3, values of bifurcational stresses and maximal values of membrane sectional forces for columns with two total lengths of 80 and 130 mm, corresponding to three symmetrical and anti-symmetrical buckling modes regarding the axis of symmetry, are presented. The ratios of values of subsequent bifurcational loads to the lowest value of the bifurcational load σ_i_/σ_4_ are included. For the TH-column of the total length 80 mm (Table 4), the lowest value of bifurcational load occurs for the symmetrical mode (S) with the number of half-waves along the longitudinal direction equal to *m* = 1S (mode 1 in Table 3). As one can easily see, values of longitudinal inner forces are significantly higher than 100 N/mm, except for the lowest value of bifurcational load σ_1_.

**Figure 14:**
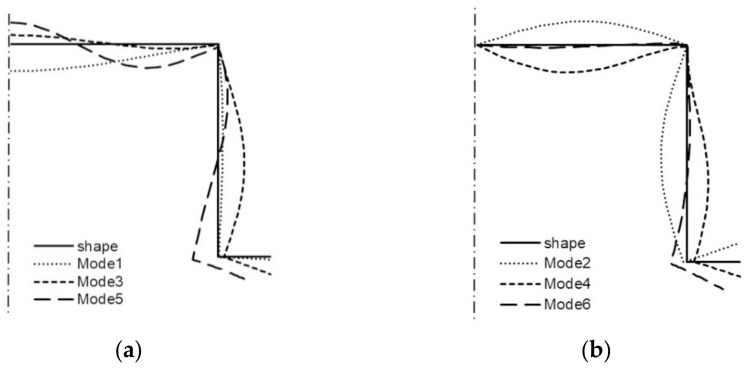
Buckling modes of the TH-channel of the total length 80 mm: symmetrical modes 1, 3, 5 in Table 3 (**a**), and anti-symmetrical modes 2, 4, 6 in Table 3 (**b**).

**Figure 15:**
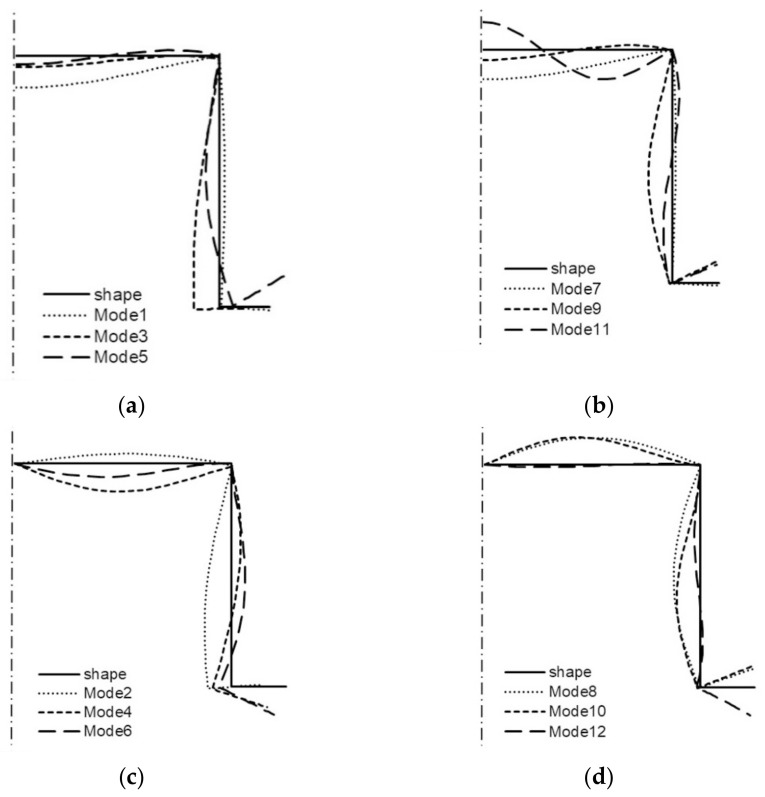
Buckling modes of the TH-channel of total length 130 mm (Table 3): symmetrical modes 1, 3, 5 (**a**), modes 7, 9, 11 (**b**), and anti-symmetrical modes 2, 4, 6 (**c**), modes 8, 10, 12 (**d**).

Buckling modes are depicted in Figure 14. For the two lowest symmetrical and anti-symmetrical buckling modes, the corners of the TH-column do not displace. For the remaining four modes, the corner joining the web with the flanges does not displace, whereas the second corner joining the web with the boundary reinforcements is subject to displacement, which corresponds to the distortional–local mode. Next, post-buckling equilibrium paths were determined, employing the SAM only and covering from a one-modal approach (i.e., *J* = 1—uncoupled buckling) up to a 4-modal approach (*J* = 4). In the calculations, identical to the channels, the amplitude of imperfections (5) was assumed: |ζ_r_^*^| = 0.2 where *r* = 1, 2, …, *J* and various combinations of their signs. Here, the post-buckling equilibrium path does not attain the maximal value. Therefore, in Table 5, for individual modal approaches for the assumed dimensionless value of shortening Δ/Δ_min_ = Δ/Δ_1_ = 5.0, dimensionless values of overloads: σ/σ_min_ = σ/σ_1_ are given. As seen, an interaction between the buckling modes under investigation is absent in practice. Attention should be drawn to the fact that for the 4-modal approach, a pair of anti-symmetrical modes was considered. While analyzing the TH-channel of the total length equal to 130 mm, it was found that the lowest value of the buckling load corresponded to the symmetrical mode of two half-waves along the column length (i.e., *m* = 2S—mode 7 in Table 3). In this table, values of bifurcational loads and maximal values of membrane forces for two types of modes and two values of half-wave numbers *m* = 1, 2 are listed. The ratios σ_i_/σ_min_ = σ_i_/σ_7_ are given as well. Values of longitudinal sectional forces are higher than 100 N/mm in 10 cases, except for two lowest values of bifurcational loads for *m* = 1 and *m* = 2. Buckling modes are presented in Figure 15. As can be easily seen, the buckling modes: symmetrical modes 3 and 5 (Figure 15a) and anti-symmetrical modes 2, 4, 6 for *m* = 1 (Table 3) are distortional modes caused by high values of longitudinal forces.

The remaining modes are the local ones, for which the corners of the TH-column cross-section do not displace practically. Like the TH-column of the length 80 mm, post-buckling paths do not attain maximal values (i.e., ultimate load-carrying capacity). Thus, in Table 4, for the value of the dimensionless shortening Δ/Δ_min_ = Δ/Δ_7_ = 5.0, the value of the load is σ/σ_min_ = σ/σ_7_. For a one-modal approach (*J* = 1), the overload is equal to 3.67, whereas for 2- to 4-modal approaches, the overload decreases approx. 1.25 times. When only an interaction of symmetrical modes is accounted for, the overload decreases to 3.08. When two additional anti-symmetrical modes are included in the 4-modal approach, the overload diminishes up to 2.94.

In further considerations, TH-columns of total length equal to 225 mm were dealt with. In Table 5, the bifurcational values of stresses, maximal values of membrane sectional forces, in brackets: the number of half-waves *m* and the symmetry conditions for the ANM, and the values of bifurcational loads for the FEM are listed. For this length, four values of numbers of half-waves m forming along the column length were analyzed. The aim was to check an interaction of modes when *m* = 1 with a larger number of half-waves of local buckling. The lowest value of the bifurcational load occurs for symmetrical mode 19 in Table 5 (i.e., σ_19_). For each number of half-waves, the values of sectional forces for three symmetrical and three anti-symmetrical buckling modes were analyzed.

The maximal bifurcational loads were attained for mode 6 when *m* = 1 (Table 5). In three cases (modes 11, 12, and 24), the FEM modes which would correspond to the ANM modes were not found. The most difficult problems consisted in assigning the FEM modes for *m* = 2, where the error in bifurcational loads reaches 28%. A very large number of degrees of freedom in the FEM causes these modes to differ significantly from the ANM “pure” modal modes. With an exception for the cases when the difference in bifurcational loads is over 10% (four cases) and in three cases lacking recording, the differences in bifurcational loads between the ANM and the FEM are lower than 7%. Among 24 modes under consideration, only for three modes (modes 7, 13, 19 in Table 5) are the values of maximal longitudinal forces lower than 50N/mm, and for 18 modes they are higher than 100 N/mm.

**Figure 16:**
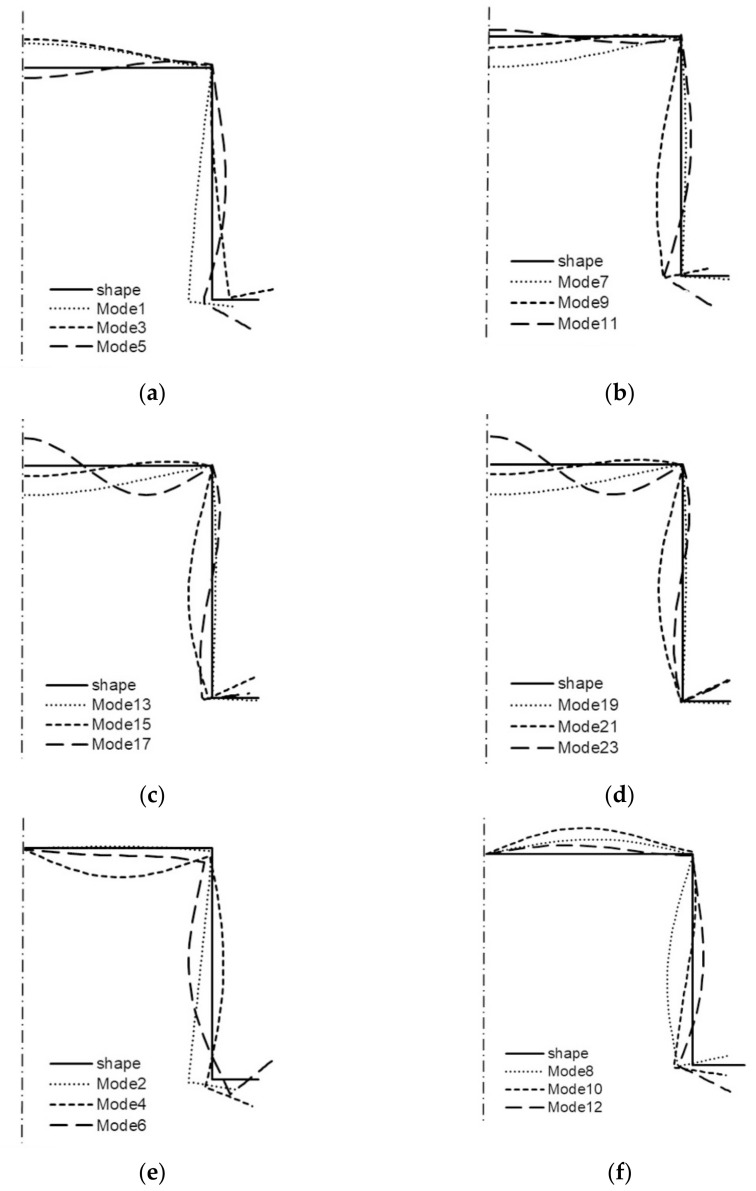

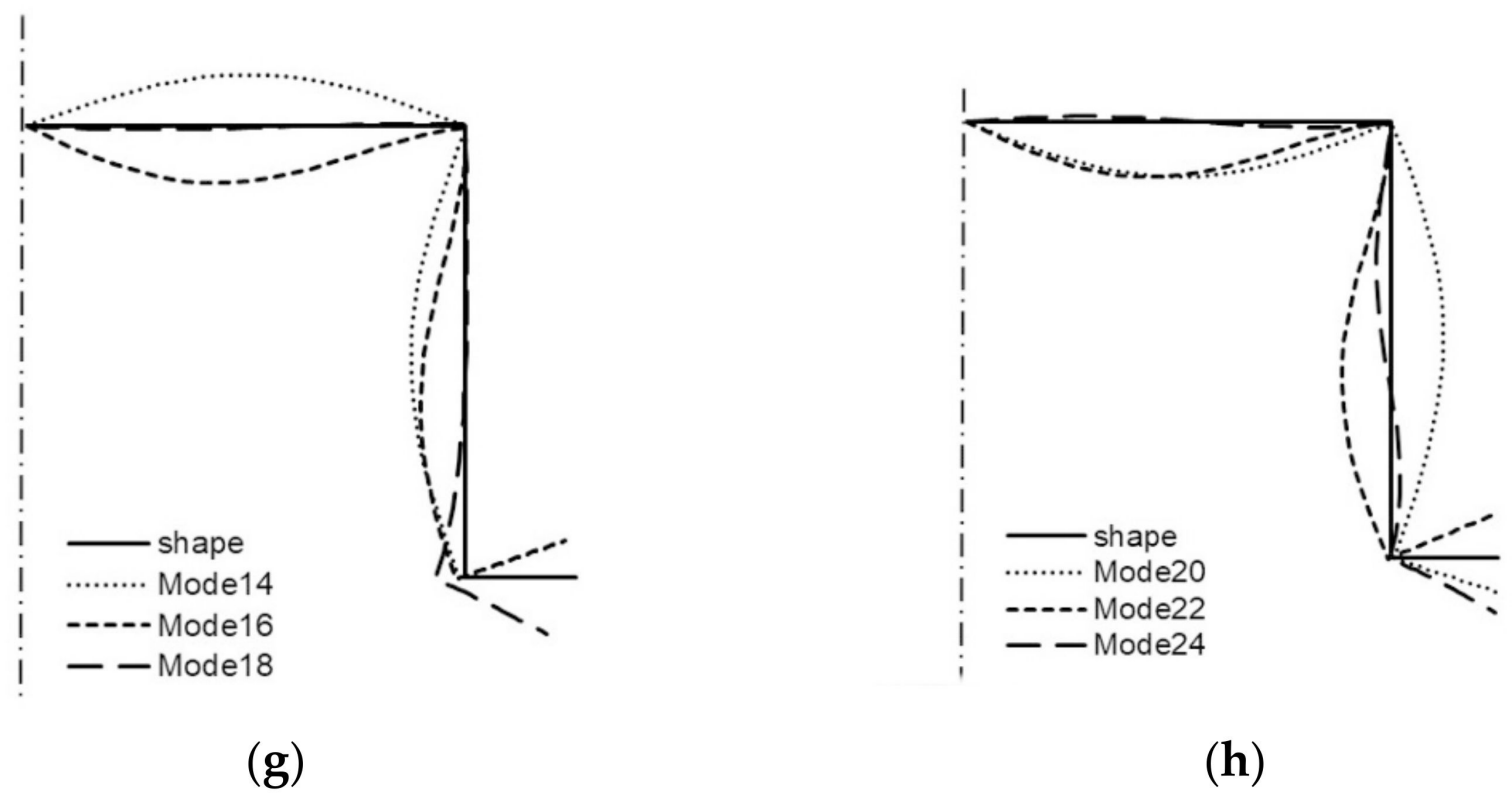
Buckling modes of the TH-column of total length 225 mm (Table 5): symmetrical modes 1, 3, 5 (**a**), modes 7, 9, 11 (**b**), modes 13, 15, 17 (**c**), modes 19, 21, 23 (**d**), and anti-symmetrical modes 2, 4, 6 (**e**), modes 8, 10, 12 (**f**), modes 14, 16, 18 (**g**), modes 20, 22, 24 (**h**).

In Figure 16a–h, buckling modes for the values of numbers of half-waves along the longitudinal direction (i.e., *m* = 1–4) for symmetrical (Figure 16a–d) and anti-symmetrical conditions (Figure 16e–h) on the axis of symmetry of the cross-section are depicted. For *m* = 1, all symmetrical modes (modes 1, 3, 5, Table 5) and anti-symmetrical modes (modes 2, 4, 6, Table 5) are distortional modes, because the corners displace along with the boundary reinforcements. Anti-symmetrical mode 2 is the only one where the corner displaces between the web and the flange. The distortional modes for *m* = 2 are modes 8–12, and for *m* = 3 only anti-symmetrical mode 18.

**Figure 17:**
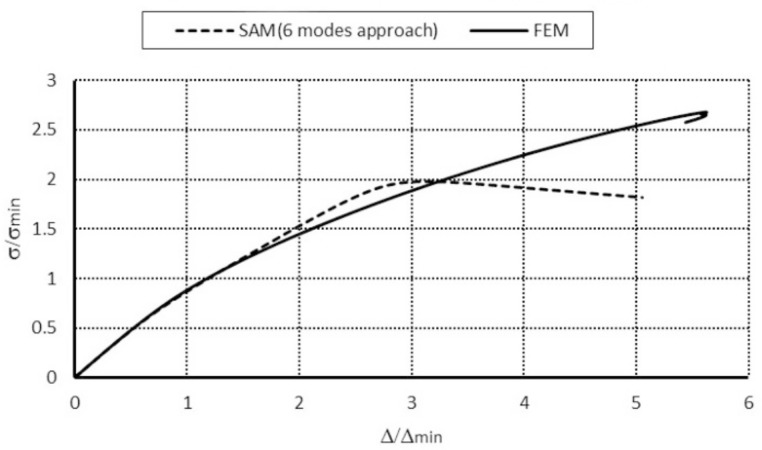
Dimensionless post-buckling equilibrium paths for the TH-channel of total length 225 mm.

**Figure 18:**
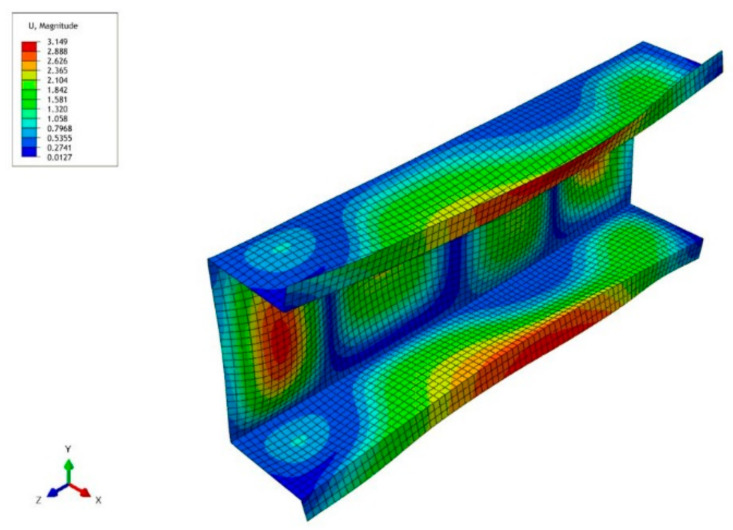
Collapse mode at the point of the ultimate load-carrying capacity for the TH-channel of total length 225 mm.

In Table 6, values of the dimensionless ultimate load-carrying capacity referring to the lowest value of the bifurcational load σ_19_ (i.e., σ_S_/σ_19_) for both the SAM and the FEM are listed. The value of the amplitude of the initial deflection was assumed as |ζ_r_^*^| = 0.2 (when *r* = 1, 2, …, *J*). An interaction of the primary mode when *m* = 1 (mode 1 in Table 5) and subsequent higher modes (i.e., modes 7–24 in Table 5, when *m* > 1) was considered. This is caused because modes of a various number of half-waves m higher than 1 forming along the length of the column under consideration (i.e., for *m* > 1) do not interact in practice. In the interaction of selected modes 1–6 when m = 1 (Table 5) and modes 19–24 when *m* = 4 (Table 5), 2–4-modal approaches were considered. The attained values of the dimensionless load-carrying capacity for these approaches differ slightly, and the lowest value of the dimensionless load-carrying capacity equal to 1.98 (Table 6) was obtained for the 4-modal approach. In Table 6 values of the dimensionless load-carrying capacity for an interaction of selected modes 1–6 when *m* = 1 (Table 5) and modes 13–18 when *m* = 3 (Table 5) are given. The lowest value of the bifurcational load σ_13_ when *m* = 3 is slightly higher than σ_19_. Here, 2–6-modal approaches were considered. With an increase in the number of degrees of freedom (i.e., for higher values of *J*), to which nonzero imperfections correspond as well, the dimensionless load-carrying capacity decreases less than 0.5%. For an interaction of selected modes 1–6 (*m* = 1, Table 5) and modes 7–12 (*m* = 2, Table 5), the lowest bifurcational value occurs for mode 7, for which the value of the bifurcational load referring to the lowest value of the bifurcational load σ_7_/σ_19_ equals 1.37. As already mentioned for modes 7–12 (*m* = 2, Table 5), in six modes under consideration, five are distortional modes, for which longitudinal inner forces are significant. In coupled interactions for modes 1–6 (*m* = 1, Table 5) and modes 7–12 (*m* = 2, Table 5), 2- and 4-modal approaches were considered. The obtained value of the dimensionless load-carrying capacity equals σ_S_/σ_19_ = 2.01. It should be taken into account that the minimal value of the bifurcational load for mode 17 (*m* = 2, Table 5) is 1.37 higher than for mode 19 (*m* = 4, Table 5), and the value of the dimensionless load-carrying capacity determined from the equations of equilibrium was recalculated to the lowest bifurcational value σ_19_. Thus, distortional–local interactions when *m* = 2 can be significant, as already noticed in [9]. In Table 6 results of the dimensionless ultimate load-carrying capacity for the SAM are compared to the FEM. Also, in this case, the SAM outcomes are lower and differ by 1.34 times. The lowest post-buckling equilibrium paths are shown in Figure 17, whereas Figure 18 presents a collapse mode corresponding to the ultimate load-carrying capacity according to the FEM. For the column of the total length equal to 225 mm, post-buckling equilibrium paths up to the value σ/σ_19_ ≈ 2 overlap (Figure 17). As seen, the buckling mode for σ_S_/σ_19_ corresponds to the coupled buckling mode 1 and 19, when *m* = 1 and *m* = 4 (Figure 18).

Finally, TH-columns of total length 330 mm were subject to analysis. Taking into consideration the conclusions for the columns of total length 225 mm, analogous to the shorter lengths of the columns, three bifurcational values for the mode with a single half-wave occurring along the column length (i.e., *m* = 1) and the lowest local modes with five half-waves (i.e., *m* = 5) for symmetrical and anti-symmetrical conditions of the axis of symmetry along with their corresponding values of inner forces, are given in Table 7 for the ANM. The values of maximal longitudinal inner forces are higher than 30 N/mm for 11 of the 12 modes under consideration, and they are higher than 100 N/mm for seven modes as well. Also, the FEM results were quoted. For the two highest bifurcational loads corresponding to the anti-symmetrical modes, the buckling mode corresponding to the SAM was not found in the FEM. The maximal differences between the values obtained with the ANM and the FEM are lower than 3.5%.

**Figure 19:**
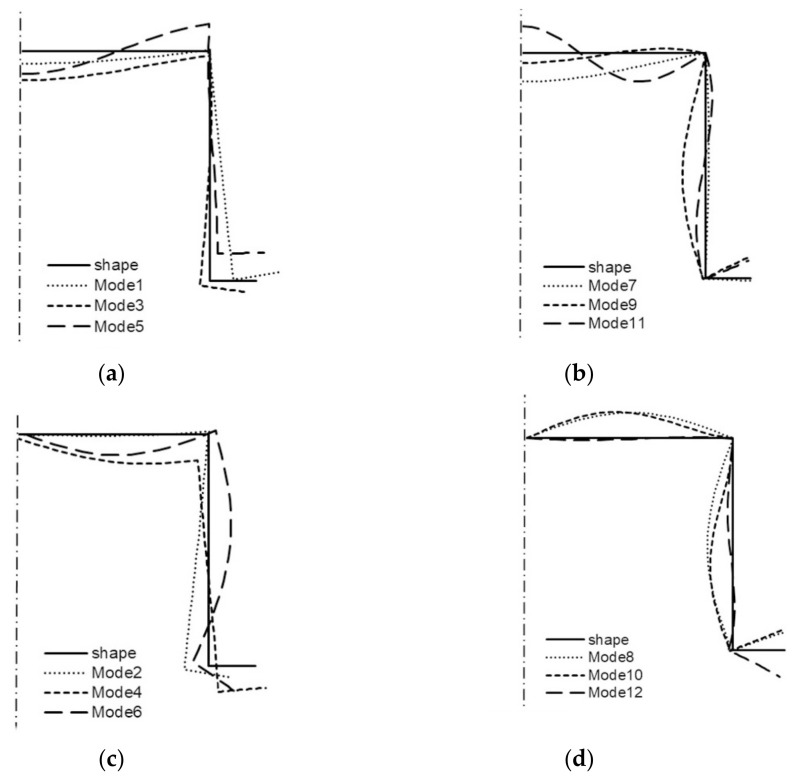
Buckling modes for the TH-channel of total length 330 mm (Table 7): symmetrical modes 1, 3, 5 (**a**), modes 7, 9, 11 (**b**), and anti-symmetrical modes 2, 4, 6 (**c**), modes 8, 10, 12 (**d**).

The buckling modes for the ANM are shown in Figure 19. As can be easily noticed, all modes 1–6 for one half-wave along the column length (i.e., *m* = 1, Table 7) are distortional modes, and for modes 7–12 (*m* = 2, Table 7), they are symmetrical only. Attention should be drawn to modes 4, 5 (*m* = 1, Table 7) and mode 11 (*m* = 5, Table 7), for which displacements of the corner joining the web with the flange are high.

In Table 8 values of the dimensionless load-carrying capacity σ_S_/σ_7_ for the SAM are given on the assumption of 2–6-modal approaches. The values of the load-carrying capacity are practically the same and equal to σ_S_/σ_7_ = 1.57, whereas for the FEM they are equal to 2.0. Identical to shorter lengths of the TH-column, the dimensionless load-carrying capacity in the SAM is lower than in the FEM. In the FEM, modes that stabilize the post-bucking path contribute. The FEM model is more prone to this than the SAM model.

**Figure 20:**
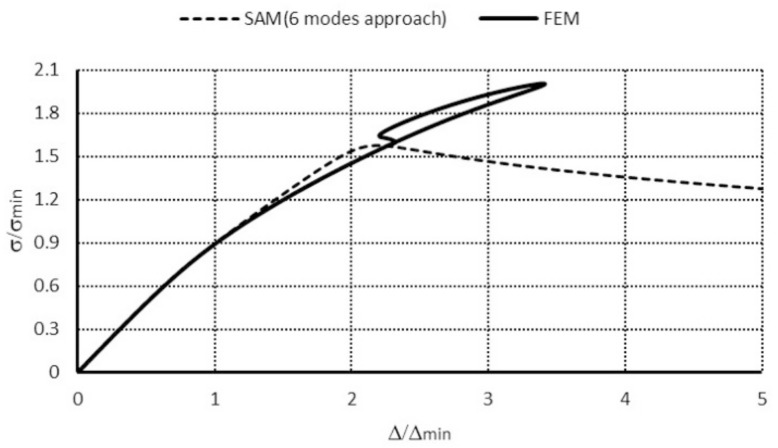
Dimensionless post-buckling equilibrium paths for the TH-channel of total length 330 mm.

**Figure 21:**
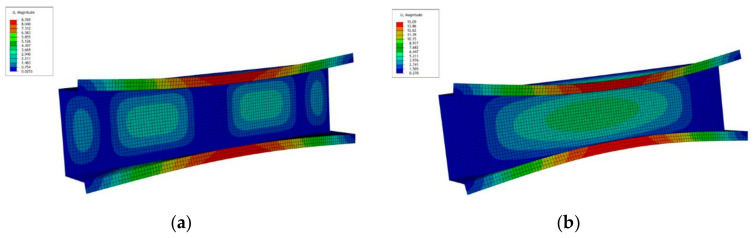
Collapse mode for the TH-channel of total length 330 mm at the moment the ultimate load-carrying capacity σ_S_/σ_7_ = 2.04 is attained (**a**), and after a jump when σ/σ_7_ ≈ 1.6 (disruption of computations) (**b**).

Figure 20 shows post-buckling equilibrium paths for the SAM and the FEM. The next figure depicts a collapse mode for the load-carrying capacity according to the FEM (Figure 21).

In Figure 20, equilibrium paths obtained from the SAM and the FEM are presented. The shape of the plots is close to the value of σ/σ_7_ ≈ 1.6. For higher values of the overload, the FEM post-buckling path grows up to the load-carrying capacity σ_S_/σ_7_ = 2.04. The most interesting is the part of the curve obtained with the Riks method, for the curve descending to the load-carrying capacity value determined in the SAM (i.e., for σ_S_/σ_7_ ≈ 1.6), then due to a loss of convergence, the FEM Abacus package stopped the computations. As opposed to the post-buckling curves in Figure 8 and Figure 9 (FEM), the descending part of the curve (Figure 20) lies outside the path regarding the ascending part. Such a curve shape is very unusual for the descending part of the path, and it requires further detailed numerical analysis. However, this does not fall within the scope of the present study.

In Figure 21a, a buckling mode for the ultimate load-carrying capacity σ_S_/σ_7_ = 2.04 is presented. Along the TH-channel length, one can see four buckling half-waves for the web, and two half-waves are very clearly visible. There is only one buckling half-wave on the flanges with the boundary reinforcements. Figure 21b shows a mode for the point when the computations were disrupted (i.e., for σ/σ_7_ ≈ 1.6). Here, one buckling half-wave occurs along the column length, both in the web and in the flanges. It indicates that a new buckling mode, which causes the column to be relieved and which leads to its destructions, appears.

## 4. Conclusions

A problem of rapid, unexpected exceeding of the load-carrying capacity was discussed using the example of hybrid thin-walled columns with open cross-sections under compression. The numerical simulations, conducted with the semi-analytical SAM based on Koiter’s theory, allowed for solving the linear eigenproblem and determining bifurcational loads, their corresponding eigenvalues, and components of membrane sectional forces. Distributing membrane sectional forces that are in balance based on the linear solution, it was possible to indicate the eigenvalue and its corresponding eigenvectors, which can affect rapid, unexpected exceeding of the load-carrying capacity of the hybrid thin-walled structure under discussion. The results were verified with the FEM in the linear and nonlinear range.

The detailed simulations were conducted for simple, supported, hybrid C-channels and TH-channels of the assumed lengths. The walls of the columns under investigation were made of two isotropic materials with a step-variable gradation of mechanical properties in the elastic range. While selecting the column lengths, the authors followed the principle that the determined maximal absolute values of longitudinal forces should attain their maxima in the solutions to the eigenproblem, which causes strong interactions between the selected eigenmodes. Summing up the results obtained for the assumed lengths and the determined maximal absolute values of membrane longitudinal forces with their corresponding buckling modes, one can see that there was a coupling between them based on the displacement of the corner for a given mode caused by a strong longitudinal sectional force. The reverse reasoning is correct as well.

In the SAM, it was possible to consider a precisely defined and finite number of buckling modes, taking into account in the interaction of these modes. This allowed one to determine the key modes that decided post-buckling equilibrium paths and the load-carrying capacity of the structure. In the FEM, it was practically impossible to choose which modes were to be considered. Including two anti-symmetrical modes in the multimodal SAM approach caused a significant decrease in the load-carrying capacity, and the stronger it was, the higher were the values of longitudinal sectional forces observed for the selected modes. The most crucial interactions of anti-symmetrical modes took place for the distortional global and local mode. In the FEM, it was not possible to confirm the results obtained when the anti-symmetrical modes were analyzed. The ultimate load-carrying capacity determined with the FEM corresponded to the SAM only if symmetrical modes were considered. Similar differences in the evaluation of the load-carrying capacity when a coupled interaction of anti-symmetrical buckling modes in the SAM multimodal approach was included, and an invisible effect of these modes on the load-carrying capacity in the FEM was found in [3].

In the FEM numerical simulations, two algorithms were employed to solve the nonlinear problem of stability loss, namely, the Riks algorithm and the Newton–Raphson algorithm. The results for stable equilibrium paths in both algorithms were identical. However, the Riks algorithm allowed us to catch the effect of a jump between two stable equilibrium paths. The post-buckling equilibrium path determined with the Riks method under low overloads was the same as that obtained with the SAM. At higher overloads, it overestimated the ultimate load-carrying capacity, then it jumped onto another equilibrium path, coming closer to the SAM path, when only symmetrical buckling modes were accounted for in the interaction. In the authors’ opinion, a jump between equilibrium paths results from the fact that new buckling modes appear. It causes the outcomes attained within both methods to become closer. These observations should be followed by further in-depth analysis.

## Figures and Tables

**Table 1:** Bifurcational stresses (i.e., σ*_i_* in MPa) and maximal absolute values of membrane forces (i.e., |*N_xi_*|_max_/|*N_yi_*|_max_|/|*N_xyi_*|_max_ in N/mm) for selected buckling modes of channels with two lengths: *L* = 210 mm and *L* = 250 mm.

Mode Number Denoted as *i*-Index	Bifurcation Parameters	*L* = 210 mm		*L* = 250 mm	
		ANM	FEM	ANM	FEM
1	σ_1_ in MPa	49.7 (1S)	49.4	60.2 (1S)	59.8
	|*N_xi_*|_max_/|*N_yi_*|_max_|/|*N_xyi_*|_max_ in N/mm	1.24/0.21/0.25	-	1.55/0.18/0.27	-
2	σ_2_ in MPa	332 (1S)	330	442 (1S)	438
	|*N_xi_*|_max_/|*N_yi_*|_max_|/|*N_xyi_*|_max_ in N/mm	19.4/1.84/4.05	-	27.6/1.85/4.92	-
3	σ_3_ in MP]	3980 (1S)	3859	3254 (1S)	3186
	|*N_xi_*|_max_/|*N_yi_*|_max_|/|*N_xyi_*|_max_ in N/mm	458/19.1/91.3	-	370/13.6/63.8	-
4	σ_4_ in MPa	35.3 (2S)	34.9	36.0 (2S)	35.6
	|*N_xi_*|_max_/|*N_yi_*|_max_|/|*N_xyi_*|_max_ in N/mm	0.68/0.50/0.27	-	0.77/0.38/0.25	-
5	σ_5_ in MPa	115 (2S)	114	145 (2S)	145
	|*N_xi_*|_max_/|*N_yi_*|_max_|/|*N_xyi_*|_max_ in N/mm	6.75/2.16/2.39	-	8.40/1.98/2.64	-
6	σ_6_ in MPa	1303 (2S)	1302	1747 (2S)	1749
	|*N_xi_*|_max_/|*N_yi_*|_max_|/|*N_xyi_*|_max_ in N/mm	4.60/9.17/2.05	-	7.02/8.83/2.44	-
7	σ_7_ [MPa]	96.5 (1A)	95.1	124 (1A)	122
	|*N_xi_*|_max_/|*N_yi_*|_max_|/|*N_xyi_*|_max_ in N/mm	3.47/0.39/0.98	-	4.71/0.36/1.10	
8	σ_8_ in MPa	1650 (1A)	1638	1580 (1A)	1553
	|*N_xi_*|_max_/|*N_yi_*|_max_|/|*N_xyi_*|_max_ in N/mm	139/5.94/24.0	-	214/4.54/30.6	-
9	σ_9_ in MPa	3054 (1A)	2968	3414 (1A)	3377
	|*N_xi_*|_max_/|*N_yi_*|_max_|/|*N_xyi_*|_max_ in N/mm	190/7.34/35.0	-	95.0/4.05/23.1	-
10	σ_10_ in MPa	48.6 (2A)	48.1	54.1 (2A)	53.6
	|*N_xi_*|_max_/|*N_yi_*|_max_|/|*N_xyi_*|_max_ in N/mm	1.52/0.71/0.80	-	1.75/0.59/0.81	-
11	σ_11_ in MPa	596 (2A)	596	785 (2A)	791
	|*N_xi_*|_max_/|*N_yi_*|_max_|/|*N_xyi_*|_max_ in N/mm	24.6/7.13/8.33	-	33.7/6.77/9.79	-
12	σ_12_ in MPa	1569 (2A)	1533	2085 (2A)	2036
	|*N_xi_*|_max_/|*N_yi_*|_max_|/|*N_xyi_*|_max_ in N/mm	31.8/11.8/15.4	-	48.3/11.5/19.5	-

**Table 2:** Absolute ultimate load-carrying capacity of channels.

Total Length *L* in mm	SAM			FEM
	*J*-Mode Approach	Mode Numbers (*i*-index in Table 1) used in SAM	σ_s_/σ_min_ = σ_s_/σ_4_	σ_s_/σ_min_ = σ_s_/σ_4_
210	3	4; 1; 2	-	1.49/1.20 *
		4; 1; 3	-	
	4	4; 1; 2; 3	1.42	
	6	4; 1; 2; 3; 8; 9	1.42	
		4; 1; 2; 3; 8; 10	0.94	
		4; 1; 2; 3; 9; 10	0.95	
250	3	4; 1; 2	2.52	1.49/1.20 *
		4; 1; 3	1.81	
	4	4; 1; 2; 3	1.39	
	6	4; 1; 2; 3; 8; 9	1.39	
		4; 1; 2; 3; 8; 10	0.90	
		4; 1; 2; 3; 9; 10	1.04	

* The Riks algorithm loses convergence (see Figure 8 and Figure 9).

**Table 3:** Values of bifurcational stresses and the corresponding maximal absolute values of membrane sectional forces for the TH-channel of two lengths (the analytical-numerical method (ANM) results).

Total Length *L* in mm	Mode Number Denoted as *i*-index	σ_i_ in MPa	|*N_xi_*|_max_/|*N_yi_*|_max_|/|*N_xyi_*|_max_ in N/mm	σ_i_/σ_min_
80	1	71.6(1S)	13.0/2.07/2.16	1.00
	2	298(1A)	150/5.69/21.9	4.16
	3	392(1S)	191/9.59/26.9	5.47
	4	553(1A)	223/11.2/30.0	7.72
	5	1403(1S)	916/30.1/124	19.59
	6	1509(1A)	586/25.7/80.0	21.07
130	1	110(1S)	33.1/1.44/3.35	1.62
	2	391(1A)	337/4.28/30.3	5.78
	3	445(1S)	373/8.34/33.3	6.58
	4	799(1A)	246/6.56/20.1	11.8
	5	1257(1S)	203/5.69/17.3	18.5
	6	1560(1A)	177/7.45/16.1	23.0
	7	67.6(2S)	9.36/2.56/1.91	1.00
	8	248(2A)	113/6.75/20.0	3.66
	9	323(2S)	140/9.81/23.8	4.77
	10	432(2A)	162/12.7/26.3	6.39
	11	1112(2S)	146/23.5/22.9	16.4
	12	1577(2A)	200/21.7/31.7	23.3

**Table 4:** Dimensionless overload coefficient σ/σ_min_ as a function of the dimensionless shortening Δ/Δ_min_ = 5.0 for TH-columns (ANM results).

Total Length *L* in mm	*J*-Mode Approach	Mode Numbers (*i*-Index in Table 3) Used in SAM	σ/σ_min_ for Δ/Δ_min_ = 5.0
80	1	1	3.70
	2	1, 3	3.66
	3	1, 3, 5	3.66
	4	1, 2, 3, 4	3.65
130	1	7	3.67
	2	7, 1	3.10
	3	7, 1, 3	3.08
	4	7, 1, 2, 8	2.94

**Table 5:** Bifurcational stresses (i.e., σ_i_ in MPa) and maximal absolute values of membrane forces in N for selected buckling modes of TH-channels with length *L* = 225 mm.

ANM			FEM
Mode Number Denoted as *i*-Index	σ_i_ in MPa	|*N_xi_*|_max_/|*N_yi_*|_max_|/|*N_xyi_*|_max_ in N/mm	σ_i_ in MPa
1	160(1S)	135/0.91/7.43	159
2	208(1A)	126/0.64/6.52	206
3	342(1S)	85.6/2.09/5.83	340
4	1632(1A)	86.7/4.75/21.2	1627
5	2801(1S)	51.4/4.85/12.2	2606
6	3036(1A)	243/10.0/47.1	2825
7	94.2(2S)	24.2/1.58/2.86	94.1
8	396(2A)	287/4.73/29.9	455
9	478(2S)	358/10.2/36.7	613
10	735(2A)	352/8.11/34.2	887
11	1168(2S)	338/9.20/33.3	-
12	1338(2A)	274/9.76/27.8	-
13	69.7(3S)	11.7/2.20/2.08	68.5
14	281(3A)	136/5.98/21.1	277
15	369(3)	171/9.51/25.7	366
16	512(3A)	199/11.7/28.4	496
17	1352(3S)	428/22.0/60.8	1294
18	1595(3A)	547/27.8/78.8	1319
19	68.8(4S)	7.59/3.04/1.76	67.8
20	225(4A)	94.9/7.52/18.8	221
21	288(4S)	119/10.6/22.8	286
22	371(4A)	139/14.0/25.6	363
23	910(4S)	90.1/25.2/15.8	927
24	1313(4A)	74.8/20.1/14.1	-

**Table 6:** Values of the dimensionless ultimate load-carrying capacity for the TH-channel of total length 225 mm (the semi-analytical method (SAM) and the finite element method (FEM) results).

SAM			FEM
*J*-Mode Approach	Mode Numbers (*i*-Index in Table 5) Used in SAM	σ_S_/σ_min_ = σ_S_/σ_19_	σ_S_/σ_19_
2	19, 1	2.00	2.61
3	19, 21, 1	2.00	
	19, 1, 3	1.99	
4	19, 21, 23, 1	2.00	
	19, 21, 1, 3	1.98	
2	13, 1	2.00	
4	13, 15, 17, 1	1.98	
	13, 15, 1, 3	1.96	
	13, 17, 1, 3	1.98	
5	13, 15, 17, 1, 3	1.96	
6	13, 15, 1, 3, 14, 2	1.95	
2	7, 1	2.05	
4	7, 9, 11, 1	2.02	
	7, 9, 1, 3	2.01	

**Table 7:** Values of bifurcational stresses and maximal absolute membrane forces for the TH-channel of total length 330 mm. ANM and FEM results.

Mode Number Denoted as *i*-Index	ANM		FEM
	σ_i_ in MPa	|*N_xi_*|_max_/|*N_yi_*|_max_|/|*N_xyi_*|_max_ in N/mm	σ_i_ in MPa
1	125(1S)	62.8/0.29/2.39	125
2	160(1A)	55.8/0.31/1.84	159
3	549(1S)	31.7/1.29/5.52	553
4	1421(1A)	157/2.13/24.4	1403
5	2939(1S)	206/4.90/39.5	2845
6	3945(1A)	73.9/4.95/28.2	-
7	67.7(5S)	9.58/2.52/1.92	66.9
8	251(5A)	115/6.65/20.1	249
9	328(5S)	143/9.75/23.9	325
10	440(5A)	165/12.6/26.5	432
11	1136(5S)	157/23.4/24.4	1099
12	1600(5A)	231/22.4/36.5	-

**Table 8:** Values of the dimensionless ultimate load-carrying capacity for the TH-channel of total length 330 mm. SAM and FEM results.

SAM			FEM
*J*-Mode Approach	Mode Numbers (*i*-Index in Table 7) Used in SAM	σ_S_/σ_min_ = σ_S_/σ_7_	σ_S_/σ_7_
2	7, 1	1.57	2.00/1.60 *
3	7, 1, 3	1.58	
4	7, 1, 3, 9	1.58	
	7, 1, 5, 9	1.63	
6	7, 1, 3, 9, 8, 2	1.58	
	7, 1, 3, 9, 8, 4	1.58	

* The Riks algorithm loses convergence (see Figure 20).

## Data Availability

Data sharing is not applicable to this article.
